# The strong influence of management factors on coccidian infections in smallholder pig farms and the first molecular identification of *Cystoisospora suis* in Myanmar

**DOI:** 10.1051/parasite/2022006

**Published:** 2022-01-28

**Authors:** Saw Bawm, Hla Myet Chel, Yadanar Khaing, Myint Myint Hmoon, Su Su Thein, Shwe Yee Win, Nyein Chan Soe, Yu Nandi Thaw, Naoki Hayashi, Mar Mar Win, Lat Lat Htun, Nariaki Nonaka, Ken Katakura, Ryo Nakao

**Affiliations:** 1 Department of International Relations and Information Technology Yezin, Nay Pyi Taw 15013 Myanmar; 2 Department of Pharmacology and Parasitology, University of Veterinary Science Yezin, Nay Pyi Taw 15013 Myanmar; 3 Laboratory of Parasitology, Graduate School of Infectious Diseases, Faculty of Veterinary Medicine, Hokkaido University Sapporo 060-0818 Japan; 4 Rector office, University of Veterinary Science Yezin, Nay Pyi Taw 15013 Myanmar

**Keywords:** Coccidian infections, *Cystoisospora suis*, Management factors, Smallholder pig farms, PCR

## Abstract

A cross-sectional study was conducted to investigate coccidian infection and associated factors in smallholder pigs, and to identify *Cystoisospora* oocysts by PCR. A total of 500 pig faecal samples from 330 smallholder farms were collected in Nay Pyi Taw, Myanmar. The faecal flotation method was used to identify *Eimeria* and *Cystoisospora* species, and oocyst counts per gram (OPG) of faeces were recorded. Oocysts were differentiated after sporulation. Oocyst DNA was subjected to ITS1-targeted *Cystoisospora*-specific PCR. The overall coccidian oocyst detection rate by microscopic was 89.0% (445/500). Among the studied samples, 74.0% (370/500) and 70.6% (353/500), were found to be positive with *Eimeria* spp. and *Cystoisospora suis* oocysts, respectively. The sequences of *C. suis* detected were 100% identical to those of *C. suis* reported from Japan, and had 99.5% resemblance to sequences from Australia and China. Weaner pigs showed the significantly highest (*p* < 0.05) OPG when compared to other age groups. The highest intensity of coccidian infection (*p* < 0.05) was found in pigs fed local feed, pigs raised on earthen floors and pigs under poor hygienic conditions. Factors such as age, breed, feed type, and housing floors were found to be significantly associated with coccidian infection (*p* < 0.05). Age, as well as management factors including floor type, feed type, and hygiene practices on the farm, had a strong influence on the occurrence of coccidian infection in pigs. This is the first study in Myanmar on coccidian infection in pigs and molecular detection of *C. suis*.

## Abstract (fr)

Une étude transversale a été menée pour étudier l’infection coccidienne et les facteurs associés chez les porcs dans des petites exploitations, et pour identifier les oocystes de *Cystoisospora* par PCR. Au total, 500 échantillons de matières fécales de porcs provenant de 330 petites exploitations agricoles ont été collectés dans la région de Nay Pyi Taw, au Myanmar. La méthode de flottation fécale a été utilisée pour identifier les espèces d’*Eimeria* et de *Cystoisospora*, et le nombre d’oocystes par gramme (OPG) de matières fécales a été déterminé. Les oocystes ont été différenciés après sporulation. L’ADN des oocystes a été soumis à une PCR spécifique à *Cystoisospora*, ciblée sur ITS1. Le taux global de détection d’oocystes de coccidies au microscope était de 89,0 % (445/500). Parmi les échantillons étudiés, respectivement 74,0 % (370/500) et 70,6 % (353/500) ont été trouvés positifs pour *Eimeria* spp. et les oocystes de *Cystoisospora suis*. Les séquences de *C. suis* détectées étaient identiques à 100 % à celles de *C. suis* signalées au Japon, et avaient 99,5 % de ressemblance avec des séquences d’Australie et de Chine. Les porcs sevrés ont montré un OPG significativement plus élevé (*p* < 0,05) par rapport aux autres groupes d’âge. L’intensité la plus élevée de l’infection coccidienne (*p* < 0,05) a été observée chez les porcs nourris avec des aliments locaux, les porcs élevés sur des sols en terre battue et les porcs dans de mauvaises conditions d’hygiène. Des facteurs tels que l’âge, la race, le type d’alimentation et les étages se sont avérés être significativement (*p* < 0,05) associés à l’infection coccidienne. L’âge, ainsi que les facteurs de gestion, notamment le type de sol, le type d’alimentation et les pratiques d’hygiène dans la ferme, ont eu une forte influence sur la survenue d’une infection coccidienne chez les porcs. Il s’agit de la première étude au Myanmar sur l’infection coccidienne chez le porc et la détection moléculaire de *C. suis*.

## Introduction

Coccidiosis is a parasitic disease that affects a variety of livestock worldwide. It is one of the most serious infections of the gastrointestinal tract, caused by various species of the Phylum Apicomplexa [3], such as *Eimeria* species, *Cystoisospora* spp., *Cryptosporidium* spp., *Sarcocystis* spp. and *Tyzzeria* spp. Although this infection is more prevalent in suckling piglets, it can also be seen in growing and finishing pigs and boars when they are transported to or kept in crowded and contaminated pens [62]. Thirteen *Eimeria* species have been described in the domestic pig (*Sus scrofa domesticus*); however, only eight *Eimeria* species and four *Cystoisospora* species are currently common [16, 17]. Although most *Eimeria* spp. infections are asymptomatic, diarrhoea, weight loss, and even death have been reported in weaned piglets [41]. While *Eimeria* spp. are not severely hazardous, *C. suis* (syn. *Isospora suis*) is pathogenic and has an impact on suckling piglets [9, 32]. Cystoisosporiasis is now one of the most common causes of diarrhoea in neonatal piglets, with high prevalence rates all over the world [32–34, 48]. Clinical manifestations include non-haemorrhagic pasty to watery diarrhoea, dehydration, rough hair coat, loss of weight and weakness [1, 24, 29, 34, 50]. This disease demonstrates very high morbidity but low mortality and does not affect evenly all piglets in the litter, resulting in lower, unequal weaning weights, and as a result, significant economic losses [29, 35, 45].

*Eimeria* and *Cystoisospora* oocysts are typically distinguished by the number of sporocysts per oocyst (two for *Cystoisospora*, four for *Eimeria*). However, determining the genus in fresh faecal samples with unsporulated oocysts may be difficult; additionally, except some species such as *E. debliecki, E. polita,* and *E. suis* [18], differentiation amongst *Eimeria* species is challenging because of intra-specific variations [46]. Morphology is commonly employed to detect *Cystoisospora* spp., and this traditional method is relatively affordable and simple [30]. Nonetheless, because of the high fat content in faeces of pre-weaned piglet and the small size of *C. suis* oocysts, microscopic identification can be challenging and insensitive, especially when oocyst numbers are low [8]. PCR methods have been used to detect coccidian species because of their sensitivity and specificity. Molecular diagnostic methods, such as PCR amplification of the *C. suis* internal transcribe spacer 1 (ITS1) region in pigs, have been established, and molecular information on this parasite has been reported in Japan, China, and Australia [19, 31, 47].

Smallholder pig farming benefits livelihoods in a variety of ways, including product income, drought insurance, emergency cash needs, household nourishment, and crop manure. It also provides direct and indirect employment opportunities for farmers throughout the world [27]. As in many developing countries, smallholder pig farming is an attractive enterprise in Myanmar, especially for those of lower socioeconomic status. Most pig farmers in Myanmar favour backyard farming since it is simple and inexpensive. Pigs are generally raised in intensive or semi-intensive farming systems with poor biosecurity [56]. The information on coccidian infection among pigs is unknown in Myanmar. The aims of the present study were therefore to conduct the survey on coccidian infection (*Eimeria* spp. and *C. suis*), to identify factors associated with coccidian infection in pigs, and to perform molecular characterization of *C. suis*, the most pathogenic species in pigs within the Nay Pyi Taw area.

## Materials and methods

### Ethics statement

Prior to the collection of samples, written informed consent was obtained from owners of the pig farm involved in this investigation. The collection of parasitological samples from pigs was approved by the Ethics Review Committee, University of Veterinary Science, and the Livestock Breeding and Veterinary Department (LBVD), Ministry of Agriculture, Livestock and Irrigation, Myanmar (Approval number: ERC/Recom/2020(7) issued on 28 February 2020).

### Study area

**Figure 1:**
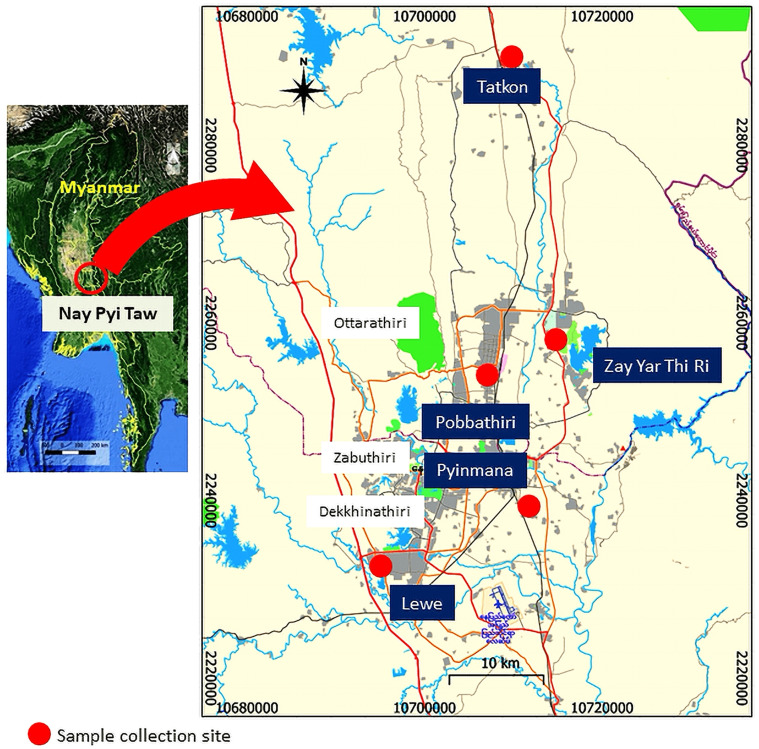
Map of Nay Pyi Taw showing sample collection locations.

A cross-sectional study was carried out in Lewe, Pobbathiri, Pyinmana, Tatkon, and Zay Yar Thi Ri Townships within the Nay Pyi Taw area, located between latitude 19° 45′ N and longitude 96° 06′ E, which is situated in the central area of Myanmar (Fig. 1). Maps were created with QGIS software version 3.14.16 (QGIS Development Team, 2020). This study was conducted between October 2020 and March 2021.

### Sample size and sample collection

As per the livestock baseline survey (2018) by the Livestock Breeding and Veterinary Department (LBVD), Myanmar, the Nay Pyi Taw area was home to 128,000 pigs in 2018. A total of 330 herds, including 53 from Lewe, 81 from Pobbathiri, 65 from Pyinmana, 51 from Tatkon, and 80 from Zay Yar Thi Ri Townships, were randomly selected for faecal sample collection (Table 1). Most of the herds surveyed were on a small scale, with an average herd size of 1–5 pigs. Only 19 and 12 herds had herd sizes of 6–10 pigs and > 10 pigs per herd, respectively. The sampling was limited to suburban and rural areas within townships that have a large number of pig farms. In total, 500 faecal samples, including 100 from each township, were collected. The samples to be examined were randomly selected regardless of their health status, age, sex and body condition. The breeds of pig were local and DYL (cross breed of Duroc, Yorkshire and Landrace). Animals were categorized as weaners (5–12 weeks), growers (> 12 weeks to 24 weeks) and adults (> 24 weeks) based on Esrony et al. [11].

**Table 1:** Number of collected samples and positive samples at each sampling location.

Studied location	Total sample		Coccidian infection (individual level)			Coccidian infection (herd level)
(Township)	Individual no.	Herd no.	All coccidian positive (%)	*Eimeria* spp. positive (%)	*C. suis* positive (%)	Positive (%)
Lewe	100	53	88 (88.0)	73 (73.0)	70 (70.0)	53 (100.0)
Pobbathiri	100	81	91 (91.0)	80 (80.0)	68 (68.0)	74 (91.4)
Pyinmana	100	65	89 (89.0)	71 (71.0)	71 (71.0)	65 (100.0)
Tatkon	100	51	87 (87.0)	72 (72.0)	68 (68.0)	46 (90.2)
Zay Yar Thi Ri	100	80	90 (90.0)	74 (74.0)	76 (76.0)	73 (91.3)
Overall	500	330	445 (89.0)	370 (74.0)	353 (70.6)	311 (94.2)

A questionnaire interview was conducted during sample collection through a prepared questionnaire, which included information on possible risk factors for coccidian infection such as history and health status of the pig, farming and feeding management system, breed, sex, age, intensive or semi-intensive, veterinary care, type of feed, water source, etc. As described in Thaw et al. [56], pigs were raised in intensive or semi-intensive farming systems in the study areas. The majority of farms in the study area had with no hygiene practices (lack of cleanliness in the pens, as well as a lack of proper disposal of sewage and animal feed waste).

### Faecal examination

Fresh faecal samples from pigs were put in plastic bags, labelled, brought to the laboratory and kept at 4 °C until processed within three days of collection. Approximately 3-5 g of faecal samples were subjected to the direct flotation method using a sugar solution (specific gravity = 1.25), as described by Zajac and Conboy [63]. Floated oocysts were examined under a light microscope and three slides from each sample were assessed for the presence of coccidian oocysts. The length and width of 20–30 randomly selected oocysts from positive faecal samples were measured at 400× magnification using a VetScan HDmicroscope (Abaxis, York, UK). Regardless of discrimination of coccidia species, calculating oocyst counts per gram (OPG) of faeces was done employing the modified McMaster technique [63]. Positive faecal samples were individually suspended in a 2.5% aqueous potassium dichromate solution (K_2_Cr_2_O_7_) in Petri dishes and left at room temperature (25 °C) for 5–7 days for oocyst sporulation [64]. After sporulation, oocysts were classified into *Eimeria* spp. and *Cystoisospora* spp., according to the number of sporocysts.

### DNA extraction

DNA was isolated from approximately 500 floated oocysts taken from 10 *C. suis-*positive samples from each township. DNA was extracted from pooled oocysts as described by Pyziel et al. [42] with slight modifications. After three washes with distilled water, the oocysts were resuspended in 50 μL of distilled water. The oocyst suspension transferred to a 1.5 mL centrifugal tube was frozen (at −80 °C in a freezer for 5 min) and thawed (at 37 °C in a water bath for 5 min), which was repeated five times. Oocysts were crushed with 0.2 mm glass beads (Biomedical Science, Tokyo, Japan), before being vortexed for 5 min at 2000 rpm [10]. A PowerFecal^®^ DNA isolation kit (MO BIO Laboratories, Carlsbad, CA, USA) was then used to extract DNA from the lysate, as per the manufacturer’s instructions. Using a Nanodrop (Thermo Fisher Scientific Inc., Waltham, MA, USA), the DNA concentration was measured.

### Molecular detection of *C. suis* by polymerase chain reaction (PCR)

The ITS1 regions were amplified by nested PCR with primer sets reported by Samarasinghe et al. [47]. The first-round PCR products were amplified using outer primers, ITSF (5′–CCGTTGCTCCTACCGATTGAGTG–3′) and EMR7 (5′–GCATTTCGCTGCGTCCTTCATCG–3′), whereas the second-round PCR products (450 bp) were amplified with inner primers, ITSGF (5′–GATCATTCACACGTGGCCCTTG–3′) and ITSR2 (5′–GACGACGTCCAAATCCACAGAGC–3′). Each reaction employed 100–200 ng of oocyst DNA in the presence of 10 μM forward and reverse primers and TksGflex DNA polymerase (1.25 U/μL) (TaKaRa Bio Inc., Shiga, Japan). Thermal cycling was initiated with denaturation at 94 °C for 1 min, followed by 40 cycles at 98 °C for 10 s, 62 °C for 15 s, 68 °C for 1 min, and a final extension at 68 °C for 5 min. The 2% Tris-acetate-EDTA (TAE) agarose gel electrophoresis was conducted to examine PCR products by staining with RedSafe Nucleic Acid Staining Solution (iNtRON Biotechnology Inc., Seongnam, Korea).

### Sequencing and phylogenetic analysis

The PCR products were excised from the gel and purified using a NucleoSpin^®^ Gel and PCR Cleanup Kit (MACHEREY-NAGEL, Düren, Germany), according to the manufacturer’s instructions. Direct sequencing of purified PCR products was performed on an Applied Biosystems 3130 Genetic Analyzer with a Big Dye v3.1 Terminator cycle sequencing kit (Applied Biosystems, Inc., Carlsbad, CA, USA). Multiple sequence alignment was done by the sequence analysis software package ATGC version 7 (GENETYX Corporation, Tokyo, Japan). The Maximum Likelihood (ML) method in MEGA X was used to evaluate phylogenetic relationships between the sequences [26]. Bootstrap analysis was done using 1000 replicates/tree. The obtained sequences were compared to those from the NCBI nucleotide database (http://www.ncbi.nlm.nih.gov/nuccore/).

### Statistical analysis

The hypothesized risk factors were examined by the Pearson Chi-square test using SPSS (version 20). Among the collected data from questionnaire interviews, factors such as age, breed, sex, farm system, and type of feed were employed for statistical analysis. Other factors, such as water source and veterinary care, were found to be consistent across farms. Differences in mean OPG in each group were analysed by ANOVA. A value of *p* < 0.05 was considered significant. The boxplots were explored by ggplot package [60], using the R language platform [43].

## Results

### Coccidian oocyst detection rate in pigs

In this study, the overall coccidian oocyst (*Eimeria* spp. and *C. suis*) detection rate by microscopic examination was 89.0% (445/500) (Table 1). Infections with coccidia were common in all study locations. Among the tested samples, 74.0% (370/500) and 70.6% (353/500) were found to be positive with *Eimeria* spp. and *C. suis* oocysts, respectively, and 55.6% (278/500) had a mixed infection. Out of the 330 herds, 94.2% (311/330) were positive for coccidian oocysts. In this study, helminth species such as *Ascaris* spp., *Oesophagostomum* spp., *Strongyloides* spp., *Trichuris* spp., *Metastrongylus* spp., *Hyostrongylus* spp., *Fasciolopsis* spp., *Paragonimus* spp., *Schistosoma* spp., and *Macracanthorhynchus* spp. were also observed (data not shown). Oocysts of *C. suis* were identified after sporulation based on the following characteristics: two sporocysts without residuum; ellipsoidal sporocysts with four sporozoites, Stieda body absent; sporocysts measuring 13.5 by 10.8 μm; observable nucleus; sporozoites measuring 10.7 by 3.9 μm (Fig. S1).

### Husbandry characteristics

In this study, commercial feeds were fed to only 9.2% (46/500) of the pigs, whereas 61.4% (307/500) and 29.4% (147/500) of pigs received local feed and a mix of commercial and local feeds, respectively. Local feed consisted of a mixture of rice bran, maize bran, groundnut cake, broken maize, etc., commonly supplemented with swills from hotels, restaurants, alcohol manufacture and their own household waste. Only a few farmers boil swill before feeding it to their animals. In order to increase the amount of feed, some farmers mix commercial feed with swill or vegetables. The housing floors were mainly earthen (78.0%, 390/500) and some were concrete/cemented (22.0%, 110/500) floors. Most of the pigs in this study were kept in farms with no hygiene practices (83.4%, 417/500) and only 16.6% (83/500) were kept on farms with hygiene practices (regular cleaning of pens, as well as proper disposal of sewage and animal feed waste). Among the studied herds, 285 herds (86.4%) received anthelmintics (ivermectin injection) at least once in a lifetime. None of the herds were given anticoccidial drugs.

### Distribution of coccidian infections amongst different age groups, feeding systems, animal housing floors and hygiene conditions

**Figure 2:**
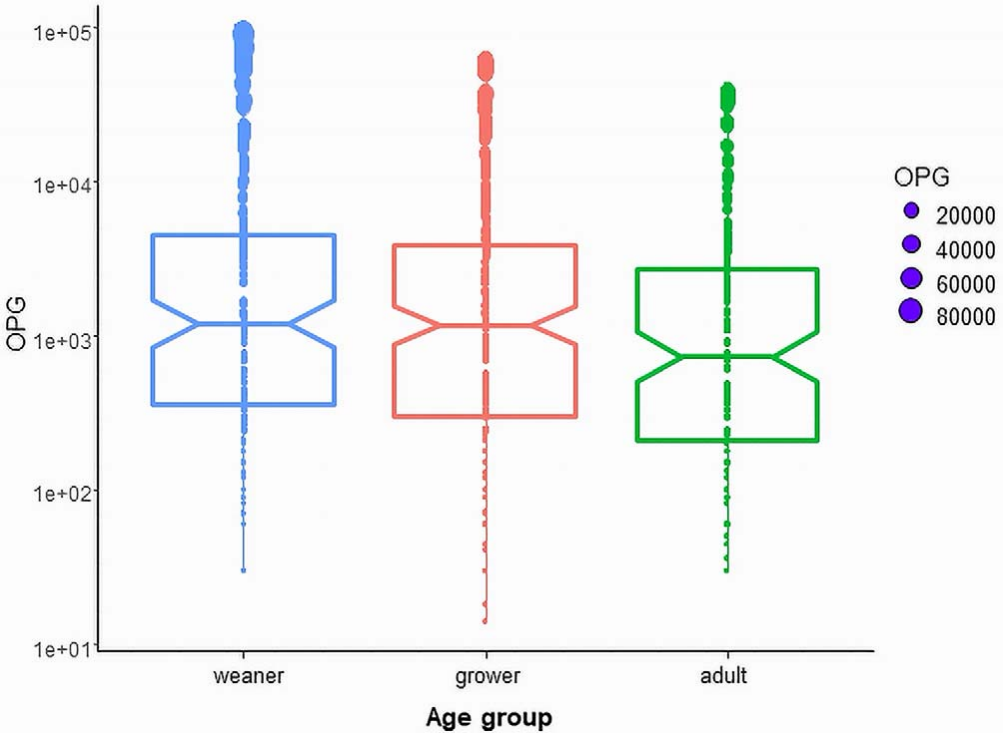
Intensity of coccidian infection (OPG) was higher in weaners than fatteners and growers (*p* < 0.05). The top and bottom horizontal lines of boxplots represent the first and third quartiles of the data range, respectively. The medians are shown by middle horizontal lines, and the data range is shown by vertical lines, with outliers plotted as points. The notches of each boxplot are approximate 95% confidence intervals of medians. OPGs are described in log number.

The mean OPG was 4,430.2 (ranging between 16.7 and 89,450) for overall coccidian infection in this study. Higher percentages of coccidian-positive samples were found in growers (93.3%) than in weaners (82.0%) and adults (90.3%). The mean OPG (Mean ± SD) values in age groups were 6,095.9 ± 13,827.8 (Median = 1200) in weaners, 4080.8 ± 8047.9 (Median = 1170) in growers and 3175.2 ± 6577.9 (Median = 735) in adults. Among age groups, weaners showed the significantly highest (*p* < 0.05) OPG when compared to other age groups (Fig. 2).

**Figure 3:**
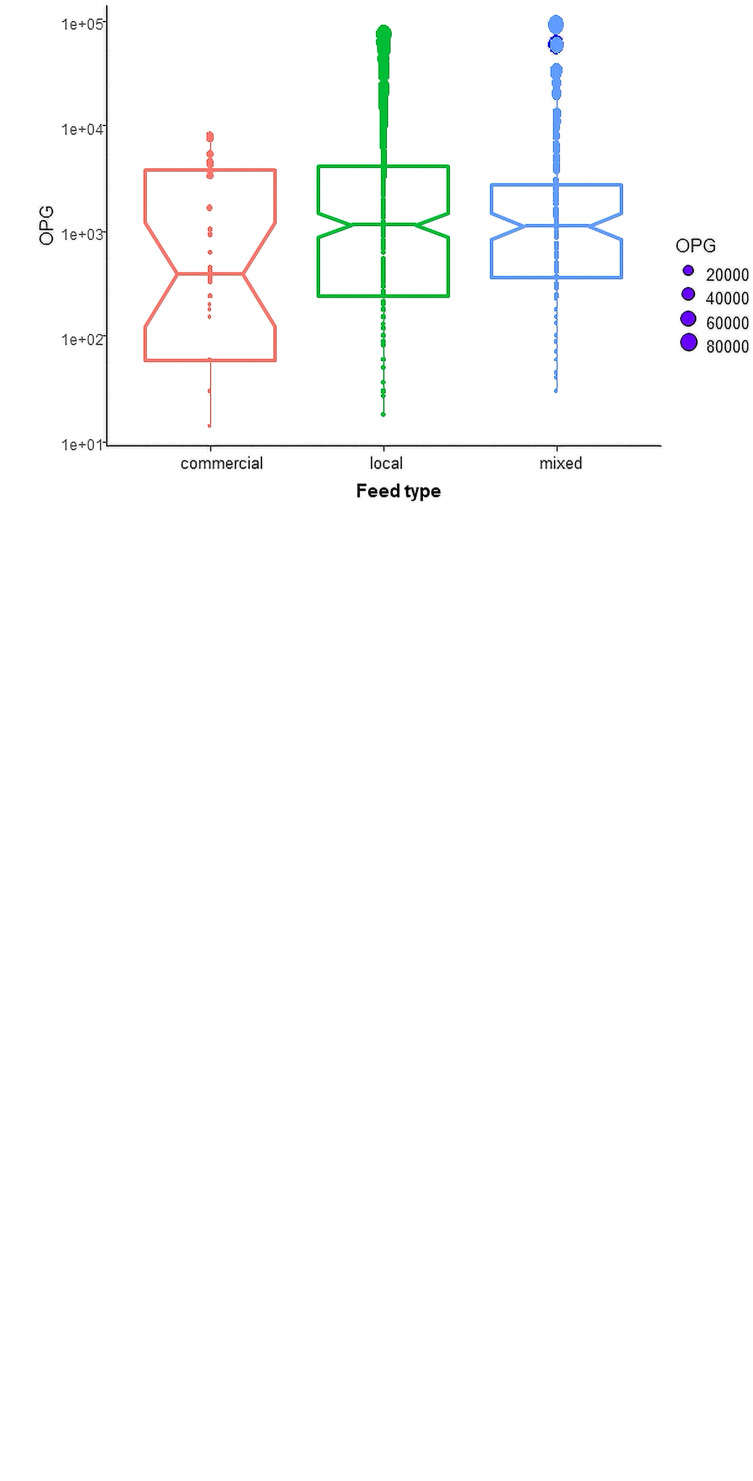
(A), (B) and (C): Intensity of coccidian infection (OPG) was lower in pigs fed with commercial feed than local and mixed feed (*p* < 0.05) (A), higher in pigs reared on earthen floors (*p* < 0.05) (B), and farms with no hygiene practices (C). The top and bottom horizontal lines of boxplots represent the first and third quartiles of the data range, respectively. The medians are shown by middle horizontal lines, and the data range is shown by vertical lines, with outliers plotted as points. The notches of each boxplot are approximate 95% confidence intervals of medians. OPGs are described in log number.

The highest intensity of coccidian infection was found in pigs fed local feed (4749.5 ± 9949.1 OPG and Median = 1140), followed by pigs fed mixed feed (4368.2 ± 10,918.4 OPG and Median = 1116), and pigs fed commercial feed (1936.4 ± 3277.8 OPG and Median = 420). The mean OPG was significantly different (*p* < 0.05) between pigs fed local feed and pigs fed commercial feed. In this study, the higher intensity of coccidian infection (*p* < 0.05) was found in farms with earthen floors (5191.2 ± 11,057.5 OPG and Median = 1320) and lower in farms with concrete floors (1989.4 ± 3764.2 OPG and Median = 435). Furthermore, significant differences (*p* < 0.05) in the intensity of coccidian infection were found between farms with no hygiene practices (5133.8 ± 10,811.7 OPG and Median = 1350) and farms with hygiene practices (1352.8 ± 2428.9 OPG and Median = 419) (Figs. 3A–3C).

### Factors associated with coccidian infection

Factors that were considered in the analysis as risks associated with parasitic infection in pigs are presented in Table 2. In this investigation, factors such as age, breed, feed type, and housing floor were found to be significantly associated with coccidian infection in smallholder pigs (*p* < 0.05). Among the age groups, growers (*p* = 0.0004, odds ratio [OR]: 3.394; 95% confidence interval [CI]: 1.677–6.869) and adults (*p* = 0.039, OR: 2.055; 95% CI: 1.026–4.118) were found to be more associated with coccidian infection than weaners (*p* < 0.05). Local breed (*p* = 0.00, OR: 5.774; 95% CI: 2.938–11.347) was found to be more associated with coccidian infection than DYL pigs (*p <* 0.05). In addition, pigs fed with local feed (*p* = 0.00, OR: 4.645; 95% CI: 2.161–9.985) and pigs fed with mixed feed (*p* = 0.009, OR: 2.823; 95% CI: 1.257–6.343) were likely to be more associated with infection than pigs fed with commercial feed, and housing with earthen floors (*p* = 0.00, OR: 3.529; 95% CI: 1.974–6.310) was found to be more associated with infection than concrete floors (*p* < 0.05). Other factors, including sex, veterinary care, water sources and hygiene practices, were not significantly associated with coccidian infection.

**Table 2:** Factors associated with coccidian infection in pigs in Nay Pyi Taw.

Factor	Total no.	Positive no. (%)	OR	(95% CI)	χ^2^	*p*-value
Age group						
Weaner	167	137 (82.0)				
Grower	198	186 (93.9)	3.394	(1.677–6.869)	12.61	0.0004*
Adult	135	122 (90.3)	2.055	(1.026–4.118)	4.25	0.039*
Sex						
Male	171	151 (88.3)				
Female	329	294 (89.4)	1.113	(0.621–1.994)	0.13	0.72
Breed						
Local	451	413 (91.6)	5.774	(2.938–11.347)	31.15	0.00*
DYL	49	32 (65.3)				
Feeding						
Commercial feed	46	33 (71.7)				
Local feed	307	283 (92.2)	4.645	(2.161–9.985)	17.82	0.00*
Mixed	147	129 (87.8)	2.823	(1.257–6.343)	6.67	0.009*
Housing floor						
Earthen	390	360 (92.3)	3.529	(1.974–6.310)	19.81	0.00*
Concrete	110	85 (77.3)				

OR, odds ratio; CI, confidence interval.

* Significant statistical findings (*p* < 0.05).

### Molecular identification of *C. suis*

**Figure 4:**
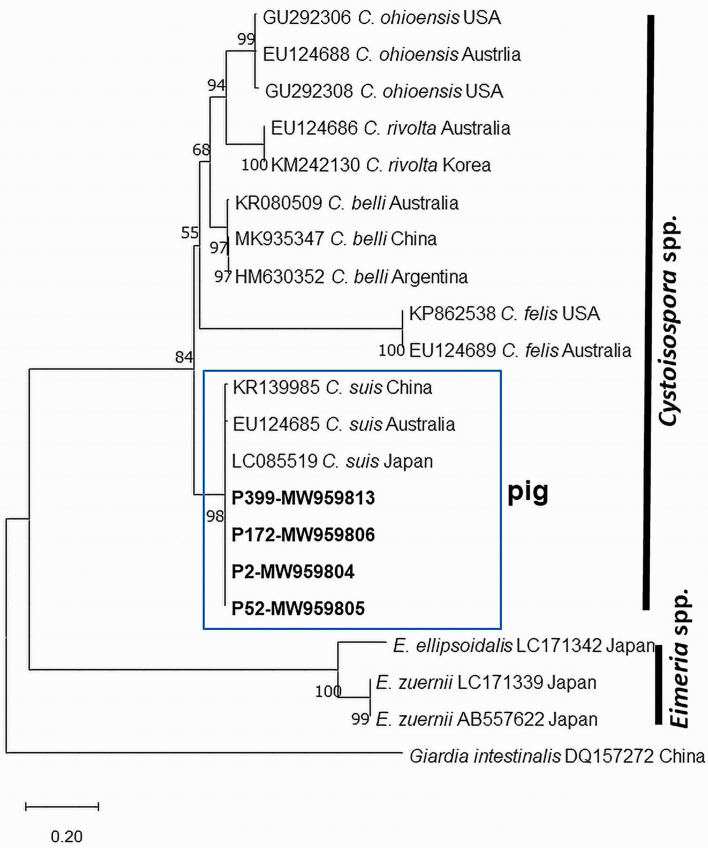
The phylogenetic relationships of partial ITS1 sequences of *Cystoisospora suis* detected in this study and reference sequences. The phylogenetic tree was constructed by the Maximum Likelihood method based on the Tamura-Nei model. The bold taxa represent the sequences obtained from the current study. GenBank accession number of each sequence is given. Bootstrap values were computed independently for the purposes of 1000 replicates.

In this study, 60 *C. suis*-positive samples (12 from each township) were randomly selected following faecal examination and PCR was done. Among them, PCR products from ten samples were amplified and the sequences then subjected to Sanger sequencing. Approximately 440-bp products of ITS1 regions were successfully amplified from oocyst DNA samples with the use of *Cystoisospora*-specific PCR and the sequences obtained were deposited in GenBank under the accession numbers MW959804–MW959813. The similarity among the ten sequences detected was 100%. The ML phylogenetic consensus tree was constructed using four representative sequences (Fig. 4) in which species of the genera *Cystoisospora* and *Eimeria* formed a well-supported monophyletic group. Sequence comparisons using BLAST revealed that the sequences obtained in this study were completely identical (100% identity) to those of *C. suis* reported from Japan (LC085519), and 99.5% identical to sequences from Australia (EU124685) and China (KR139985) (Table S1), and clustered together with all *C. suis* sequences derived from pigs. It was clearly different from those of *Cystoisospora* spp. from other hosts species.

## Discussion

We were unable to perform molecular analysis of *Eimeria* spp. in this study due to laboratory limitations. Furthermore, due to oocyst similarities, we were unable to differentiate between species by microscopic examination. Therefore, only *C. suis*, the most pathogenic species in pigs, was identified by molecular methods at this time.

Coccidian infections in pigs occur all over the world and are caused in post-weaned pigs by more than one *Eimeria* species at the same time, whereas suckling piglets normally harbour only *C. suis* [9, 64]. In this investigation, a considerably high proportion (89.0% at the individual and 94.2% at the herd level) of pigs was infected with coccidian parasites (74.4% *Eimeria* spp. and 71.0% *C. suis*). The overall prevalence of coccidian infection (*Eimeria* spp. and *C. suis*) was higher than previously reported prevalence worldwide, which ranged from 8.6% to 66.0% [4, 9, 21, 59, 64]. Similar findings were reported at the herd level, with infection rates ranging from 83% to 100% in Australia [33, 37] and 88% in the West Indies [20]. The prevalence of coccidian infection in pigs at the herd level was particularly high in Myanmar, while prevalence ranges between 8.1% and 70.0% in other countries [2, 33, 49, 64]. This variation could be due to the differences in pig husbandry practices among the study areas.

The infection intensity was found to be the highest in the weaner group although the infection rate was lower than in other age groups. It is well documented that the severity of infection in nonclinical cases of coccidiosis can be determined by counting the number of oocysts (OPG) discharged in the stool [39, 40]. In this survey, OPG was evaluated for each faecal sample. According to the Chi-square test, growers and adult pigs were more likely to be infected with coccidian infection (particularly *Eimeria* infection) than weaners in this study. Adult pigs may have a greater opportunity to come into contact with oocysts than young animals, which may explain high infection rates in growers and adult pigs. The presence of a high number of coccidian oocysts (both *Eimeria* and *Cystoisospora* spp.) in the faeces of weaned pigs has been associated with post-weaning stress and may be substantially associated with age [23, 38]. In very young piglets, the disease often takes a serious course, while age resistance leads to primarily subclinical infections in weaned animals [61]. Only a few piglets had diarrhoea during sample collection, whereas all grower and adult pigs had normal faeces. Adult pigs, despite having a lower OPG, are important reservoirs of coccidia in herds, as they excrete oocysts with infectious capability. According to Bangoura and Daugschies [5], clinical coccidiosis accounts for a relatively minor proportion of economic losses, with subclinical infections causing the highest losses. Furthermore, due to the rapid spread of these infections within a herd, particularly *C. suis* oocysts reaching the infective stage faster than *Eimeria* spp. [18, 28], as well as the resistance and long-term survival of infectious oocysts in the environment, coccidiosis is a constant threat to animal health and an economic burden on the farmer.

If a large number of oocysts are ingested, the coccidian parasite, particularly *C. suis,* multiplies in small intestine enterocytes, causing catarrhal to fibrinonecrotic enteritis with intestinal villi shortening and fusion, non-haemorrhagic diarrhoea, and reduced weight gain. This is primarily due to damage to the villi and surface area of the small intestine, which impairs proper nutrient absorption. Additionally, *C. suis* has the potential to alter the intestinal epithelium and gut microbiota, resulting in decreased nutrient absorption [22, 50]. Studies in gnotobiotic piglets suggested that *C. suis* is the primary pathogen [13], while co-infections with bacteria (e.g. *E. coli* and *Clostridium*) or viruses (e.g. Rotavirus and Coronavirus) may result in more severe clinical symptoms and mortality rates [58]. In general, the morbidity of *C. suis* infection is high, but the mortality rate is low [17].

Microscopy approaches are less accurate for quantifying oocysts in samples with mixed infections, especially when screening for a large number of unsporulated oocysts. The benefit of PCR in general is that it can be applied at any stage of the parasite and can help to discriminate coccidia species in unsporulated stages [46]. In this investigation, we employed PCR with species-specific primers to identify *C. suis* in selected samples and partial ITS1 sequences were identified. We constructed a phylogenetic tree of the ITS1 regions from various species using *C. suis* sequences deposited in GenBank (Fig. 4). Consequently, the isolate in the present study clustered with *C. suis* identified from other investigations into the same clade. The phylogenetic tree in this study reveals that *Cystoisospora* from pigs and other hosts share a common evolutionary history and may have descended from a common ancestor.

Environmental factors play a critical role in the dispersion and prevalence of coccidian infections in susceptible animals. Management factors such as feeding method, type of housing floors and hygiene conditions on pig farms may influence some differences in the occurrence of coccidian infections in pigs. According to the Chi-square test, feed type is significantly associated (*p* < 0.05) with coccidian infection in pigs in this investigation. Furthermore, higher intensities of coccidian infection (*p* < 0.05) were found in pigs fed with local and mixed feeds than in pigs fed with commercial feeds. Since *Eimeria* and *Cystoisospora* spp. are food-borne parasites, the infection is mostly spread through the consumption of contaminated feed and water containing coccidia oocysts. In the current study area, the swill utilized to feed pigs consisted of kitchen waste and by-products. To kill contaminated pathogens, this type of pig feed should be boiled at 100 °C for 1 h [36]. However, in this study, only a few farmers boiled swill before feeding it to their animals. Feeding unboiled pig feed might favour an increase in infection rates. This type of feedstuff may be infected with oocysts, and it may also be deficient in nutritional value for pigs. Pigs fed commercial feed, on the other hand, were well-nourished and met the nutritional requirements of the host. Many feed additives included in commercial feed may have a potential to improve the integrity of the intestinal epithelium, increased disease resistance and enhanced growth promotion [57]. It has been shown that the nutritional status of the host can affect the development of parasites, and it is widely believed that well-nourished animals are more resistant to parasitism than malnourished animals [12].

Another important factor is the type of flooring in pens. In this study, pigs kept on farms with earthen floors, usually under a semi-intensive system, were found to be significantly associated with coccidian infection (*p* < 0.05). Furthermore, there appeared to be a higher prevalence of coccidian oocysts in pigs raised on an earthen floor and higher OPG was also detected in pigs from an earthen floor (*p* < 0.05). Pigs kept on the ground have frequent contact with animal faeces, increasing the risk of parasite infection in the herd [6, 55]. Earthen floors, the most common form of housing floor type, undoubtedly provide a better environment for the development of parasites than concrete floors. Most parasite oocysts and eggs require adequate amounts of moisture, temperature, and humidity to develop and become infective [54], and these parameters are more likely to be reached on earthen floors. Therefore, raising on an earthen floor was positively associated with coccidian infection. The use of concrete floors with better hygiene conditions on farms, on the other hand, resulted in a lower frequency of parasites. In the study area, the majority of local breeds were kept on earthen floors and DYL pigs were kept on concrete floors. For this reason, pigs of local breed were found to be significantly associated with coccidian infection in this investigation (*p* < 0.05).

The presence of coccidian oocysts in pigs is regarded as an indicator of a farm’s hygiene status; the lower the degree of hygiene, the more frequently coccidian infections occur [18]. The coccidian infection was associated with higher OPG in pigs raised on farms with no hygiene practices when compared to pigs raised on farms with hygiene practices (*p* < 0.05). In the study area, farms with no hygiene practices are common, which favours the occurrence of coccidian infections. Following that, oocysts can easily contaminate animal feed and water. Reduced environmental contamination by thorough cleaning can be beneficial in preventing or delaying initial infections in very young suckling piglets, allowing for the development of innate resistance in littermates, hence limiting disease spread in affected animals [52]. Because oocysts are resistant to most anticoccidial drugs [33, 35], eradication is almost impossible once they have been introduced on the farm. Appropriate cleaning and disinfection, as well as sufficient time between batches to allow the pen to dry completely, are all important management practices that help limit parasite survival and spread [44]. The reduction or inactivation of infectious oocysts is critical for controlling coccidian infections and preventing early parasite exposure in piglets. Furthermore, efficient hygiene techniques, such as steam cleaning and the use of an anticoccidial disinfectant, should be implemented [53].

There are currently no vaccines available, and toltrazuril is the only medicine available for metaphylaxis that effectively suppresses oocyst excretion and improves piglet health both in the laboratory [15] and in the field [25]. However, because of the recent demonstration of toltrazuril resistance problems [51], effective hygiene management practices are also necessary for the control of coccidian infections, including *C. suis* infections [14, 53].

## Conclusions

This study is the first study in Myanmar on coccidian infection in pigs and molecular detection of *C. suis*. A high infection rate of coccidian oocysts was detected in pigs in this study area. The age of the pigs, as well as management factors such as the type of flooring, type of feeding and hygiene practices on the farm, had a strong influence on the occurrence of coccidian infection in pigs. Furthermore, coccidian infection in piglets should be given greater attention because coccidiosis, particularly cystoisosporiasis, is likely to predispose the piglet to secondary bacterial and viral infections, increasing morbidity, mortality, and management expenses [7]. Therefore, in order to manage piglet diarrhoea in Myanmar, it is necessary to pay attention to the high infection rate of coccidian species in pigs. In the future, investigations on *Eimeria* spp. of pigs in Myanmar will need to continue, using both microscopic and molecular approaches. The findings in this study provide baseline information that could be used to develop an effective control programme for pig coccidiosis on smallholder farms.

## Conflicts of interest

The authors declare that there are no conflicts of interest regarding the publication of this paper.

## Supplementary Materials

The Supplementary materials of this article are available at https://www.parasite-journal.org/10.1051/parasite/2022006/olm**Figure S1(A), (B) and (C):**
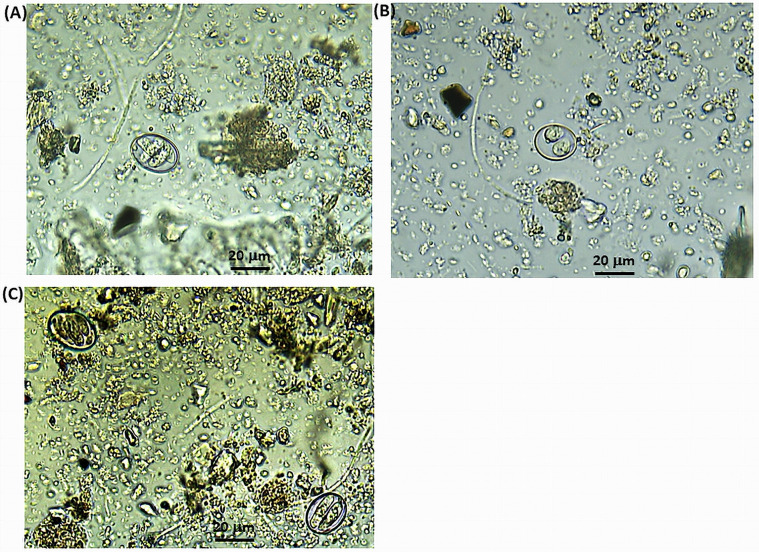
*Eimeria* spp. (A), *Cystoisospora* spp. (B) and mixed infection with *Eimeria* spp. and *Cystoisospora* (C), detected in this study.*Table S1*: Nucleotide sequence homologies (×100%) between the ITS1 genes of *C. suis* parasites from Myanmar and other *Cystoisospora* species.
