# Supplementary material for: The strong influence of management factors on coccidian infections in smallholder pig farms and the first molecular identification of Cystoisospora suis in Myanmar
Source: Parasite. 2022 Jan 28;29:1. doi: 10.1051/parasite/2022006 (PMC8796702; doi:10.1051/parasite/2022006)
Supplement: Supplementary file 1 — Table S1: Nucleotide sequence homologies (×100%) between the ITS1 genes of C. suis parasites from Myanmar and other Cystoisospora species. [file parasite-29-1-s1.pdf]

Table S1.

	Features of *Cystoisospora* spp.	1	2	3	4	5	6	7	8	9	10	11	12	13	14	15	16	17
1	P2-MW959804																	
2	P52-MW959805	1.000																
3	P172-MW959806	1.000	1.000															
4	P399-MW959813	1.000	1.000	1.000														
5	LC085519_*C. suis*_Japan	1.000	1.000	0.998	1.000													
6	KR139985_*C. suis*_China	0.995	0.995	0.995	0.995	0.991												
7	EU124685_*C. suis*_Australia	0.995	0.995	0.995	0.995	0.953	0.842											
8	KR080509_*C. belli*_Australia	0.823	0.823	0.823	0.823	0.790	0.641	0.633										
9	MK935347_*C. belli*_China	0.815	0.815	0.793	0.815	0.819	0.708	0.686	0.913									
10	HM630352_*C. belli*_Argentina	0.815	0.815	0.793	0.815	0.819	0.787	0.760	0.964	1.000								
11	GU292306_*C. ohioensis*_USA	0.708	0.708	0.688	0.708	0.719	0.650	0.588	0.691	0.750	0.829							
12	EU124688_*C. ohioensis*_Austrlia	0.710	0.710	0.710	0.710	0.710	0.703	0.704	0.809	0.809	0.809	0.992						
13	GU292308_*C. ohioensis*_USA	0.708	0.708	0.688	0.708	0.719	0.655	0.592	0.694	0.750	0.829	0.991	0.987					
14	KP862538_*C. felis*_USA	0.351	0.351	0.351	0.351	0.351	0.358	0.343	0.409	0.381	0.381	0.320	0.322	0.337				
15	EU124689_*C. felis*_Australia	0.351	0.351	0.351	0.351	0.351	0.358	0.343	0.409	0.381	0.381	0.320	0.322	0.337	1.000			
16	EU124686_*C. rivolta*_Australia	0.732	0.732	0.732	0.732	0.732	0.723	0.720	0.806	0.807	0.807	0.814	0.814	0.814	0.288	0.288		
17	KM242130_*C. rivolta*_Korea	0.738	0.738	0.731	0.738	0.731	0.721	0.724	0.842	0.848	0.848	0.847	0.844	0.843	0.308	0.308	1.000	
